# Missing signposts on the roadmap to quality: a call to improve medication adherence indicators in data collection for population research

**DOI:** 10.3389/fphar.2013.00139

**Published:** 2013-11-07

**Authors:** Bradi B. Granger, Shelley A. Rusincovitch, Suzanne Avery, Bryan C. Batch, Ashley A. Dunham, Mark N. Feinglos, Katherine Kelly, Marjorie Pierre-Louis, Susan E. Spratt, Robert M. Califf

**Affiliations:** ^1^Department of Nursing, Duke Translational Nursing Institute, Duke University Medical CenterDurham, NC, USA; ^2^Department of Health Intelligence and Research Services, Duke University and Health SystemDurham, NC, USA; ^3^Department of Medicine, Duke Clinical Research Institute, Duke University Medical CenterDurham, NC, USA; ^4^Department of Community and Family Medicine, Duke University Medical CenterDurham, NC, USA; ^5^Duke Translational Medicine Institute, Duke University Medical CenterDurham, NC, USA; ^6^Division of Endocrinology, Metabolism and Nutrition, Department of Medicine, Duke University Medical CenterDurham, NC, USA; ^7^Division of Endocrinology, Department of Medicine, Duke University Medical CenterDurham, NC, USA

**Keywords:** medication adherence, data model, process matrix, taxonomy, health behavior, self-management, secondary use, cardiometabolic

## Abstract

**Purpose:** Poor adherence to prescribed medicines is associated with increased rates of poor outcomes, including hospitalization, serious adverse events, and death, and is also associated with increased healthcare costs. However, current approaches to evaluation of medication adherence using real-world electronic health records (EHRs) or claims data may miss critical opportunities for data capture and fall short in modeling and representing the full complexity of the healthcare environment. We sought to explore a framework for understanding and improving data capture for medication adherence in a population-based intervention in four U.S. counties.

**Approach:** We posited that application of a data model and a process matrix when designing data collection for medication adherence would improve identification of variables and data accessibility, and could support future research on medication-taking behaviors. We then constructed a use case in which data related to medication adherence would be leveraged to support improved healthcare quality, clinical outcomes, and efficiency of healthcare delivery in a population-based intervention for persons with diabetes. Because EHRs in use at participating sites were deemed incapable of supplying the needed data, we applied a taxonomic approach to identify and define variables of interest. We then applied a process matrix methodology, in which we identified key research goals and chose optimal data domains and their respective data elements, to instantiate the resulting data model.

**Conclusions:** Combining a taxonomic approach with a process matrix methodology may afford significant benefits when designing data collection for clinical and population-based research in the arena of medication adherence. Such an approach can effectively depict complex real-world concepts and domains by “mapping” the relationships between disparate contributors to medication adherence and describing their relative contributions to the shared goals of improved healthcare quality, outcomes, and cost.

## Introduction

For patients with chronic illnesses such as diabetes mellitus and cardiovascular disease, poor adherence to prescribed medicines is associated with increased rates of hospitalization, serious adverse clinical events, and death (Balkrishnan, 2005; Ho et al., 2006; Bitton et al., 2013). Preventable cardiometabolic complications such as retinopathy, myocardial infarction, heart failure, peripheral neuropathy, and amputation are on average 50% more likely to occur in patients who struggle with medication adherence (Sokol et al., 2005; Tjia et al., 2010; Chen et al., 2013; Egede et al., 2013). Healthcare costs associated with poor adherence range from $100 billion to $300 billion annually (Sokol et al., 2005; National Council on Patient Information and Education, 2007; Bowry et al., 2011), contributing significantly to the projected 20% of U.S. gross domestic product to be spent on healthcare by 2020 (Leape et al., 2009).

Despite more than 4 decades of educational, behavioral, and communication-based interventions to improve adherence, a majority of patients are non-adherent to one or more prescribed medicines (Sabate, 2003; Fischer et al., 2010; Davies et al., 2013; Kyanko et al., 2013). Significant intellectual and financial investment in research to improve adherence has not yielded measureable improvements in medication adherence at the population level, and adherence-related clinical outcomes have not appreciably changed. This relative lack of progress may be due in part to issues surrounding the data. The complexity of contributing factors, variability in data definitions, and inconsistency in terms used in practice and research prevent the successful scaling of interventions from controlled research settings to real-world populations (Heisler et al., 2010; Pladevall et al., 2010; Bosworth et al., 2011). Measurement and data capture from the many entities that contribute to management of adherence over time, including patients, caregivers, providers, communities, and health systems, are inaccessible or absent (Sanfelix-Gimeno et al., 2013). The purpose of this article is to describe medication adherence data capture in a population-based intervention, the Southeastern Diabetes Initiative (SEDI), which aims to improve healthcare quality, outcomes, and costs for patients with diabetes mellitus. Based on our experience in this use case, we posit that developing a data model and using a process matrix to redesign data collection for medication adherence will improve variable identification, data access, and future research on medication-taking behaviors.

## Approach

### Use case: the southeastern diabetes initiative (SEDI)

The Southeastern Diabetes Initiative (SEDI) (http://innovation.cms.gov/initiatives/Health-Care-Innovation-Awards/North-Carolina.html) is a population-based intervention designed to address healthcare quality, outcomes, and costs for patients with diabetes. Using community and clinic-based clinical teams in four counties in the southeastern United States (Durham and Cabarrus Counties in North Carolina, Mingo County in West Virginia, and Quitman County in Mississippi) we aim to: (1) improve healthcare delivery for individual and population-level diabetes management; (2) improve outcomes and quality of life for diagnosed and undiagnosed adults with type 2 diabetes; and (3) reduce overall healthcare costs for populations with type 2 diabetes by reducing hospital and emergency department admissions, improving access through non-physician providers and technology-enabled consultations, and providing interventions focused on medication management and adherence.

### Use case problem statement

Identifying variables for medication adherence is critical to achieving improvements in quality, outcomes, and costs in SEDI. Because lack of adherence among diabetes patients is associated with costly hospitalizations, serious adverse clinical events, and death (Sokol et al., 2005; Tjia et al., 2010; Chen et al., 2013; Egede et al., 2013), identifying measurable indicators of medication adherence and management of medications over time was an important step in evaluating the work of the clinical team to improve the quality of healthcare delivery.

At project initiation, we faced the challenge of developing data collection to record behaviors and interventions associated with medications. We assumed that the existing electronic health record (EHR) systems used by our clinical teams would be adequate to collect information associated with medications and interventions within the project. However, while developing our data requirements, we realized that existing EHR data collection did not capture the granularity necessary for analysis. We therefore designed an independent data collection system that complemented EHR data collection for each of the four participating counties.

In the following sections, we discuss our rationale for this decision and our experience designing data collection for the intervention.

### Requirements analysis

We analyzed data requirements for each of the pre-specified indicators of medication adherence and management of adherence. These included documentation of variables associated with the steps in medication reconciliation, shared goal setting, and planning for management of medications between consultation intervals. Measures for each of these data requirements were selected from observable, objective data sources, as well as from patient-reported, subjective measures such as the Morisky Medication Adherence Survey (MMAS) (Morisky et al., 2008). Each data requirement was selected and designated for a measurement frequency that correlated with meaningful intervention time points. Measures such as the MMAS, Patient Activation Measure (PAM-13), health literacy assessment (REALM-SF), and self-care skills assessment for managing medications will be collected at baseline and at follow-up at intervals of 6 months. In each of the four counties participating in SEDI, these variables were not available in the EHR. We determined that any effort to incorporate these forms of data capture into existing EHR systems would be costly and not feasible within the project timelines.

### Development work

A multidisciplinary working team met regularly for 5 months to develop the data collection design for a stand-alone electronic data capture system to be used in conjunction with existing EHR systems. The team comprised clinical providers (physicians, nurses, nutritionists, licensed clinical social workers, and community health educators), data analysts, and programmers. Consensus was sought for parsimonious variable selection that would achieve the goal of high-quality, accessible, and robust measures for medication adherence. After the design was reviewed by clinical teams in all four counties, it was finalized in February 2013.

**Figure 1:**
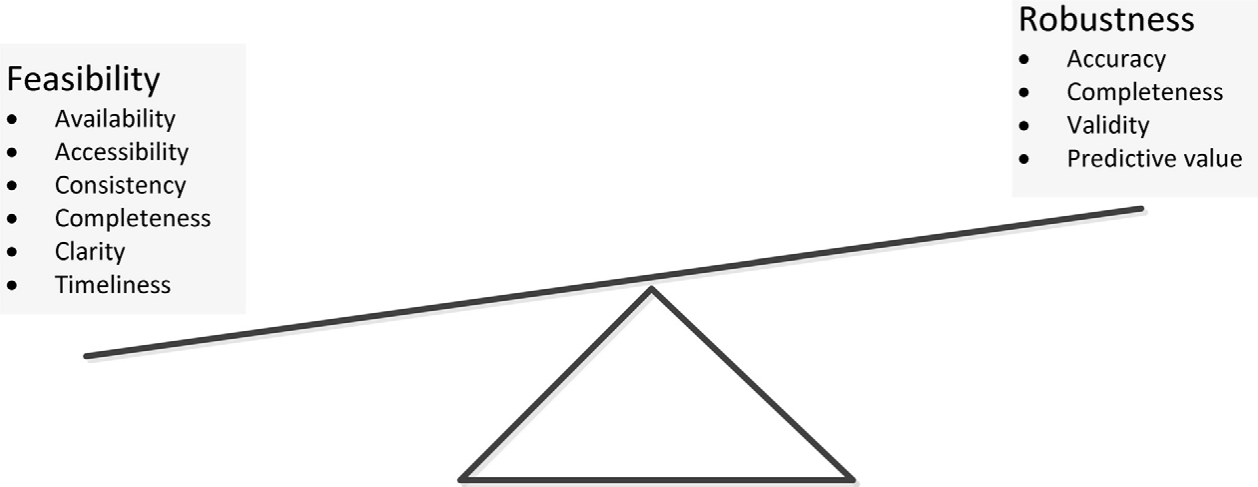
**Data properties for medication adherence data elements**.

We encountered many decision points during these meetings that proved important to variable selection and affected project development. The decisions centered on trade-offs between choosing highly robust variables (e.g., accuracy, validity and predictive value of the data elements) and choosing variables that were feasible to obtain (data availability, accessibility, consistency, completeness, clarity, and the timeliness of data capture) (Figure 1). Often, the most robust measures were not collected at the SEDI sites as part of usual care and therefore were not reflected in the EHR. This led to feasibility challenges, including the cost and time commitment required to complete some patient-reported outcome (PRO) surveys. In addition, many interventions and strategies used to support medication adherence take place in outpatient settings governed by multiple regulatory agencies, leading to inconsistent definition of terms and selection of measures.

To apply consistent decision-making criteria, a list of data selection and developmental decision points was compiled and used as a process matrix. We applied judgments within the process matrix to make decisions about data quality, access, and the feasibility of data collection processes for medication adherence (Table 1A). We then kept a similar list of decision points and judgments for variable selection and data documentation issues that would ultimately affect the analysis plan (Table 1B). The criteria were applied to variable or measure for medication adherence across each of the patient care interactions. Most decision points were resolved using the clinical, regulatory, data analytics, and research expertise offered by group members. Because of the unique multidisciplinary composition of our group, we were able to engage in constructive discussions that resulted in redirection of data element selection and lessons learned.

**Table 1A:** **Process matrix: data collection considerations for medication adherence variable selection**.

**Data of interest**	**Data element considerations**	**Feasibility score (n/7)**	**Robustness score (n/5)**
Duration of data collection	Longitudinal registry, all conditions	✓	na
	Longitudinal, but with specific focused hypothesis		
	Longitudinal registry for specific disease		
Scope of medications of interest	Study drug only- investigational agent	na	✓ (prescribed drugs only)
	Specific class of medication only	✓		
	All prescribed medications	na		
	“PRN” or “take as needed” meds	na		
	“OTC” or “over the counter” meds	na		
	Add vitamins, supplements	na		
Data source-medications of interest	Patient reported drug names	✓	Poor accuracy
	Verification by pill bottles	✓		
	Computerized physician order entry	✓		
	Pharmacy records of fills	✓		
	Pharmacy claims (e.g., Medicaid)		
Dosing specificity	Timing (time of day)	✓	Poor accuracy
	Frequency (x/day, weekly, other)	✓		
	Dose (mg, tablet, cc)	✓		
	Taken as directed—with food/without	✓		
	Route (by mouth, subcutaneous, etc)		
Measure of prescription fill rate	Patient reports filling medication	✓	✓
	Pharmacy reports fill information	✓	✓		
	Claims data reports	✓	✓		
	Add stops and restarts	na		
Mode of adherence measure	Patient reported	✓	Poor accuracy
	Provider observed clinical judgment	na		
	Pill bottle counts	na		
	Pharmacy refills	na		
	DOT (e.g. HIV/TB)	na		
	Serum drug level	na		
	Pill chips	na		
	Bottle lid opens (MEMS cap)		
Level of data required	Binary (adherent/ not adherent)	✓	✓
	80% or 100% threshold	na		
	Categorical (< 20; 21–80%; > 80%)	na		
	Likert scale (1–5)	✓	✓		

**Table 1B:** **Process matrix: considerations for analysis limitations for medication adherence data**.

**Data of interest**	**Data options present in the dataset**	**Limitation present**	**Solution selected**
Generalizability	One cohort/one condition	Y/N	[name item]
	More than one cohort/one condition		
	Multiple conditions		
	Multiple locations, one system		
	Multiple health systems		
Longitudinal assessment intervals	One-time measure only		
	Planned intervals of assessment (e.g. every 6 months)		
	Regular + unscheduled contacts		
	Irregular intervals (e.g. every patient contact; no planned)		
Medication data element codification	Free text with later use of text mining		
	Free text with later formal medical coding processes		
	Combinations of free text/codification		
	Codification at time of data entry (FDB, other schemes)		
Medication management support variables:codification	Diffuse message/educated (importance of taking drug)		
	Condition-specific message (diabetes drugs)		
	Agent-specific education/message (HIV drugs)		
	Provider consultations (recommend insulin adjustments)		
	Address physical barriers (transport, cost)		
	Address social barriers (patient perception)		
Data collection timing and mode	Prospective (case report form) vs. retrospective		
	Secondary dataset—electronic or chart abstraction		
	Direct data collection (real-time data collection/entry)		
	Hybrid approach in combining both secondary and direct data capture		
Analysis plan for medication adherence patterns	Intention to treat analysis approach		
	Patient is on drug on <date>		
	Measure patient-time-on-drug as being intervals between report dates (do not assess stops/restarts)		
	Full-grain detail of stops/restarts		
Intended analysis objectives for outcomes	Drug categories only (HTN, diabetes)		
	Trends for reduction in dose/frequencies		
	Trends in adherence/compliance		
Observational bias	Case controls?		
	Moving to randomization scheme?		
	Adding other sources?		
	Combining active and passive data collection?		
	Modifying source of observational data collection (EHR modifies source systems at Duke)		
Data incompleteness	Accept less power in analyses		
	Imputation		
	Increase number of patients		
	For active data collection, implement processes for incomplete data alerts		
	For active data collection, increase efforts to “backfill” data (site queries, manual chart abstraction)		

### Application to clinical intervention and case study

In the following scenario, we present a hypothetical case study to provide context for the decisions made regarding data capture in the SEDI project. This case highlights common patient concerns, provider quandaries regarding medication management, and caregiver and health system opportunities to support patients in the daily work of taking medication-taking. The case also highlights important opportunities for data capture, many of which go unrecognized and unfulfilled—resulting in missed chances to garner fact-based feedback for all participants in the process of care.

John Doe is a 62-year-old male with uncontrolled type 2 diabetes, hypertension, and hyperlipidemia. His medication regimen includes metformin 1000 mg twice daily, insulin glargine (Lantus) 20 units via subcutaneous injection at night, lisinopril 20 mg daily, and atorvastatin (Lipitor) 40 mg daily (Table 2). John Doe lives alone in an apartment, is currently uninsured, and does not have personal transportation.

**Table 2:** **Example patient data for SEDI MEDICATION LOG data collection**.

**Drug name**	**Indication**	**Dose**	**Unit**	**Frequency**	**Route**	**Is patient judged to be taking medication as directed at least 80% of the time?**
**MEDICATION DATA COLLECTION FOR SEDI PARTICIPANT JOHN DOE ON JANUARY 15, 2013**						
METFORMIN	Type 2 diabetes	1000	MG	twice a day	By mouth (PO)	〇Yes
						⊠ No
						〇 Unable to assess
LIPITOR	High LDL cholesterol	40	MG	Once per day	By mouth (PO)	⊠ Yes
						〇 No
						〇 Unable to assess
LISINOPRIL	High Blood Pressure	20	MG	Once per day	By mouth (PO)	⊠ Yes
						〇 No
						〇 Unable to assess
INSULIN, Lantus	Type 2 diabetes	20	Units	Once daily	Subcutaneous injection	⊠ Yes
						〇 No
						〇 Unable to assess

John Doe and a family member were present during his initial home visit. Mr. Doe reported adherence to his medication regimen. During inspection of pill bottles, both the lisinopril and atorvastatin bottles were found to have been refilled within the past month and the appropriate number of pills were left in the bottle. However, the bottle of metformin had too many pills left in the bottle and the box of insulin pens sitting on the kitchen table had expired. The patient stated that his daughter was able to help him with the cost of the medications for this month, but also anticipated that he would be unable to pay for the next month's supply because his daughter is only present for a short visit.

On further questioning, the patient reported frequently forgetting to take his metformin at night. He stated he is less likely to forget his insulin because he kept the box of insulin on the kitchen table to remind him to take it. While demonstrating insulin administration, the patient injected insulin into his abdomen but removed the pen early.

The provider recommended prescribing extended-release metformin for once-daily dosing to increase adherence (Table 3). The community health worker was asked to help the patient submit a Medicaid application and to help him use public transportation to obtain medications. The patient was educated regarding insulin storage, insulin administration, and reviewing insulin expiration dates.

**Table 3:** **Example patient data for ACTION PLAN data collection**.

**Intervention description**	**Intervention primary focus**	**Intervention primary type**	**Intervention begin date**	**Intervention closure date**	**Intervention success**	**Optional intervention comments**
**ACTION PLAN FOR SEDI PARTICIPANT JOHN DOE ON JAN 15, 2013, GENERATED THROUGH DISCUSSION BETWEEN MR. DOE, MR. DOE'S DAUGHTER, AND SEDI CLINICAL TEAM**						
Recommend Metformin dose adjustment to PCP	⊠ Provider	⊠ Medication	1/15/2013	1/18/2013	⊠ High	Dr. Smith from clinical team contacted Mr. Doe's PCP, Dr. Jones, who concurred with Metformin dose adjustment, and submitted ePrescription to Mr. Doe's pharmacy
	〇 Patient	〇 Behavior			〇 Moderate
	〇 Family member/caregiver	〇 Referral to medical resource			〇 Low
		〇 Referral to non-medical resource		
Address cost of Metformin	〇 Provider	〇 Medication	1/15/2013		〇 High	Mr. Doe's application for Medicaid services has not been submitted. Referred Mr. Doe to Community Health Worker for assistance in filing application
	⊠ Patient	〇 Behavior			〇 Moderate	
	〇 Family member/caregiver	〇 Referral to medical resource			〇 Low
		⊠ Referral to non-medical resource		
Address transportation barrier for pharmacy pick-ups	〇 Provider	〇 Medication	1/15/2013		〇 High	Mr. Doe's daughter has had trouble picking up Mr. Doe's prescriptions at pharmacy. Referred Mr. Doe's daughter to Community Health Worker for assistance in bus route scheduling.
	⊠ Patient	〇 Behavior			〇 Moderate	
	〇 Family member/caregiver	〇 Referral to medical resource			〇 Low
		⊠ Referral to non-medical resource		
Address drug expectations (side effects; symptom response)	〇 Provider	⊠ Medication			⊠ High	
	⊠ Patient	⊠ Behavior			〇 Moderate
	⊠ Family member/caregiver	〇 Referral to medical resource			〇 Low
		〇 Referral to non-medical resource		
Address any needed supports for drug administration	〇 Provider	⊠ Medication			⊠ High	
	⊠ Patient	⊠ Behavior			〇 Moderate
	⊠ Family member/caregiver	〇 Referral to medical resource			〇 Low
		〇 Referral to non-medical resource		
Address plan for drug accommodation or adaptation of daily life	〇 Provider	⊠ Medication			⊠ High	
	⊠ Patient	⊠ Behavior			〇 Moderate
	⊠ Family member/caregiver	〇 Referral to medical resource			〇 Low
		〇 Referral to non-medical resource		

As illustrated in this use case, adherence to oral medication can be assessed using pill bottle fill dates, pill counts and devices such as MEMs caps. However, assessing adherence to an injectable drug such as insulin poses unique challenges. In the example above, delivering a dose of insulin requires knowledge of injection technique, and withdrawing the needle prior to finishing injection can result in delivery of less than a full dose of medication. Additionally, injecting into areas of lipohypertrophy (areas of fibrosis or lipid tissue deposits) can lead to less absorption of the insulin. Alternating sites of injection from areas of slower absorption, such as the thigh, to areas of quicker absorption, such as the upper arm or abdomen, can lead to variations in insulin bioavailability. Some insulin regimens require multiple shots per day that must be taken at certain times with regard to meals. Insulin expiration dates and storage are additional variables that are difficult to assess outside of the patient's home.

In this clinical case, although prescribed medications were obtained and initiated, the implementation and management of the medications was insufficient without considerable support from family caregivers, providers, and community-based resources. Data capture for the variables reflecting management of adherence was also found to be insufficient.

### Gap analysis and specification of adherence taxonomy constructs

Our data collection design related to medication adherence relied heavily on the combined expertise of the team rather than existing sources. We found that although some measures were well-defined, such as the MMAS, we lacked definitive sources and exact data element specifications to define all variables within our project, especially to support appropriate measures relating to medication adherence and leading to robust analyses of the intervention. We relied on the adherence taxonomy (Vrijens et al., 2012) for a critical conceptual basis. This taxonomy comprises two overarching constructs: *Adherence to Medications* and *Management of Adherence*.

The World Health Organization (WHO) has defined medication adherence as “the extent to which a person's medication-taking behavior corresponds with the prescribed therapeutic regimen” (Sabate, 2003). Yet for researchers attempting to measure and evaluate these behaviors in relation to clinical outcomes, this broad definition entails a conundrum of choices and decisions regarding variable selection, with each option potentially varying widely across studies. As a result of this variability, the ability to standardize data elements in an EHR, case report form, or data warehouse is lost and opportunities to scale interventions or generalize study findings across settings and populations are sacrificed. The taxonomy for medication adherence creates fresh possibilities for both research and practice (Vrijens et al., 2012). Developed in a series of international consensus panels hosted by the European Society for Patient Adherence, Compliance and Persistence (ESPACOMP), the taxonomy was defined and validated using an established Delphi technique carried out by the Ascertaining Barriers to Compliance (ABC) Project from 2009 to 2012. The methodological processes of these combined efforts have been well described (Vrijens et al., 2012) and have resulted in international consensus and validation of terms that define two overarching constructs of medication adherence: *Adherence to Medication* and *Management of Adherence* (Table 4).

**Table 4:** Summary of the taxonomy and definitions of medication adherence^*^

**Taxonomy**	**Definition**
Adherence to medications	The process by which patients take their medications as prescribed, composed of initiation, implementation and discontinuation.
	*Initiation* occurs when the patient takes the first dose of a prescribed medication.		
	*Discontinuation* occurs when the patient stops taking the prescribed medication, for whatever reason(s). Implementation is the extent to which a patient's actual dosing corresponds to the prescribed dosing regimen, from initiation until the last dose.		
	*Persistence* is the length of time between initiation and the last dose, which immediately precedes discontinuation.		
Management of adherence	The process of monitoring and supporting patients' adherence to medications by health care systems, providers, patients, and their social networks.

* From Vrijens et al. (2012).

Importantly, this taxonomy distinguishes between the work associated with taking the prescribed medication (*Adherence to Medication*) and the work that supports management of the medication (*Management of Adherence)*. The former refers to initiating the medication, persisting in taking the medicine over time, and discontinuing the medicine at the appropriate time. The latter encompasses prescribing accurately, facilitating accessibility, and ensuring the understanding of instructions for administration. The need to establish these two distinct areas that both contribute to a shared goal is at the heart of achieving the goals of improved quality, clinical outcomes, and costs. For this reason, we propose that applying this taxonomy to identify EHR variables would advance research in this field and improve data collection.

### Data domains, elements, and definitions for adherence to medication

The taxonomic construct *Adherence to Medication* comprises four domains: *initiation, implementation, discontinuation*, and *persistence*. Their distinct representative data elements provide multiple options for measuring, assessing, and evaluating each distinct domain.

Table 5A shows data elements within each of the four domains of *Adherence to Medication*. Data elements are measureable components of variables, such as “medication name,” “dose,” “frequency of administration,” and “duration” of prescribed medication dosing. The definition of a discrete data element is constant, but the availability of data elements that represent a variable of interest in a given study context may vary. For example, to measure and evaluate the variable “medication adherence” in a clinical efficacy trial, the required data elements might include “medication name” (i.e., the specific study drug), “dose,” “frequency of administration,” and “duration of dosing interval (time until prescribed titration to next dosing interval).”

**Table 5A:** **Taxonomy domains and element definitions: adherence to medication**.

**Data domains**	**Data elements**	**Data capture metric**
Initiation	Pharmacy data for fill date—Medication possession ratio (MPR)	MPR; PDC; MEMS
	Pharmacy data for refills—Proportion of days covered (PDC)	DOT; F/U call data point		
	Medication electronic monitoring systems (MEMS caps)		
	Directly observed therapy (DOT)—hospital dispensed at discharge		
	Patient-reported start date—follow up call questions		
Implementation	Medication electronic monitoring systems (MEMS caps)	MEMS
	Patient-reported adherence-Morisky medication adherence scale (MMAS survey)	MMAS		
Discontinuation	Pharmacy data for refill frequency—prescription stop date	MPR; PDC
	Patient-reported stop date	F/U call data point		
Persistence	Self-report; MPR; PDC	TEACHBACK; *MMAS; MPR; DEDUCE*

For the domain *initiation*, defined as the time at which the first dose of a medication is taken, commonly used data elements include “prescribing date” or “prescription fill date.” Each has unique limitations in terms of accessibility, reliability, and validity. For instance, prescription fill date offers an accessible surrogate for initiation, but its reliability and validity with regard to actual initiation of medication-taking are limited. Other, more sensitive data elements in this domain include MEMS electronic cap removal time stamps, blister pack documentation, or self-report; each has its respective advantages and limitations. However, an organizational framework or data model can formalize a repository of data elements and standard data definitions for each element and provide a framework for critical thinking to determine the most robust and accessible data elements for a given research question in a given study context.

In contrast, some research questions require less granularity in data element definition. To measure and evaluate “medication adherence” as an indicator of quality care in a CMS healthcare redesign evaluation, selected data elements need only reflect the guideline-based class of drugs (e.g., beta blocker), as opposed to a specific medication within that class, such as labetalol. The medication need not be further specified or defined by generic versus brand name, and the dose, route, and frequency of doses per day may not be important data elements to capture in this context.

In each of these examples, the discrete data elements required to achieve a valid, reliable measure of medication adherence are different. But in each case the need to select one or more representative data elements from each key domain remains a critical step in strengthening the study design. By considering the context in which information will be used, investigators and practitioners can make informed decisions regarding what data elements are needed to offer support and feedback or to draw a conclusion about medication adherence. The formalized data model provides a map for prospectively considering data element selection instead of relying on a *post-hoc*, arbitrary approach for determining which data elements might be accessible and available for use. By using a taxonomy-based data model to assess study design and data element availability, researchers will be able to prospectively estimate lack of access and account for this before the analysis and evaluation phases of a project.

### Data domains, elements, and definitions for management of adherence

The taxonomy described in Vrijens et al. (2012) does not specify data domains for *Management of Adherence*, as it does for *Adherence to Medications*. *Management of Adherence* as a unique taxonomical component is defined as “the process of monitoring and supporting patients' adherence to medication by health care systems, providers, patients and their social networks” (Vrijens et al., 2012, p. 697). It can therefore be characterized by the domains of self-management and support for self-management, including support provided by healthcare systems, health policy, communities, providers, caregivers, and patients' social networks. In addition, “shared goal setting” as it relates to medications is an implicit domain within the construct from both a provider and patient-centered perspective. Examples of the data domains and possible data elements representing each are shown in Table 5B. These data domains and their respective data elements must be further defined, integrated, and validated if application of the taxonomy is to yield meaningful, patient-centered measures and describe feasible roles for families, providers, and health systems.

**Table 5B:** **Taxonomy domains and element definitions: management of medication**.

**Data domains**	**Data elements**	**Data capture metric**
Self-management	Attend appointments: A1c measurement and lipid levels	Self-reported surveys of self-care: DSCI; DSME; SCHFI; MMAS
	Self-monitor: blood glucose, blood pressure, weight (med-related fluid balance)		
	Use reminder strategies: pillbox, logs, technology-based reminder alarms, watches	MPR; PDC		
	Pharmacy reported prescription fill/refill rates		
Provider support	Listen; explain; support behaviors; communicate feedback (specifically regarding labs/logs/reported symptoms or side effects)	HCAHPS-item #	*CTM #1-3*
	Client Centered Care Questionnaire (CCCQ)	CAHPS		
		*HL item 1-4*		
		*PCMH items x-z*		
		CCCQ		
Caregiver support	Emotional; tangible; informational; companionship	Surveys/self-report/interviews
Health system support	CMS 5-star rating	HEDIS metrics / fill / refill rates
	HCAHPS – Coleman CTM-3	Surveys (Likert scale)		
	CAHPS – PCMH & HL items;		
	Behavioral Economics-based med adherence incentives		
Social network support	On-line/live group participation; frequency of network engagement	Interview/text fields	Network participation counts

### Methodology for instantiating the data model through a process matrix

**Figure 2:**
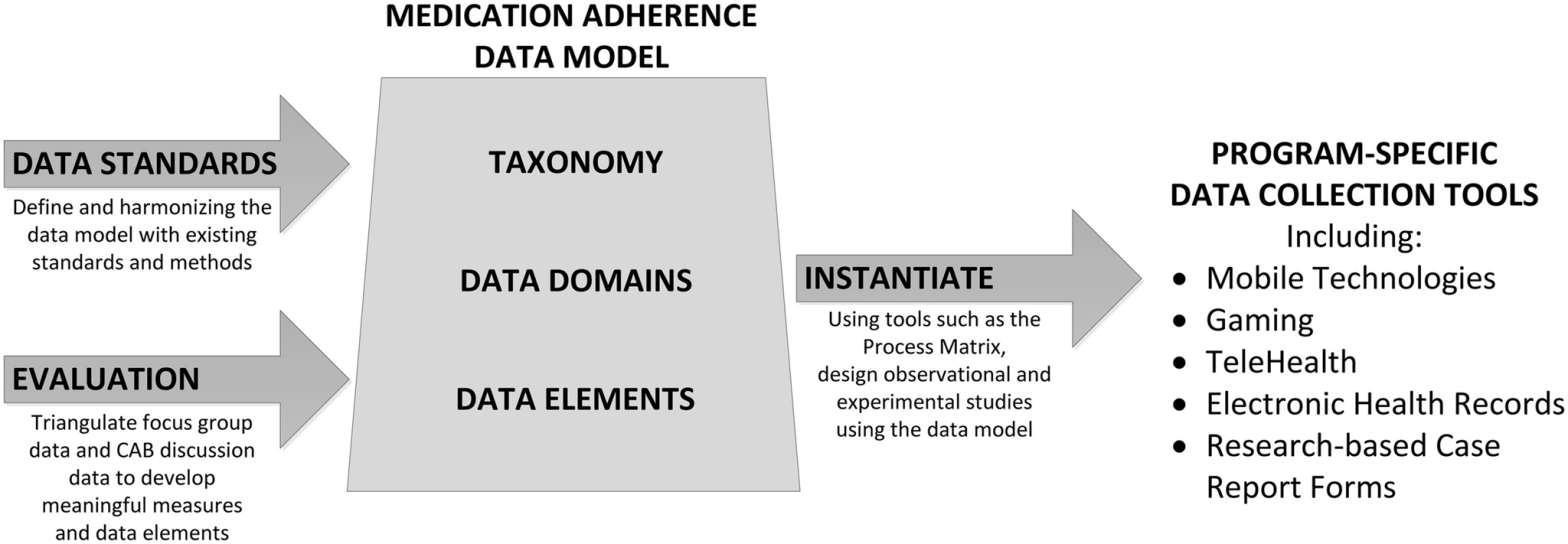
**Proposed data model**.

Using the taxonomy as a guide to specify desirable variables within each of the two major constructs, we defined a process for application and implementation. Initial steps for applying the taxonomy include: (1) defining data domains, or groups of related data elements for two taxonomic constructs: *Adherence to Medication* and *Management of Adherence*; (2) identifying data elements, including definitions and attributes, within each data domain; (3) aligning data elements with international standards; and (4) instantiating the taxonomy into use (i.e., applying and evaluating the performance of data domains and data elements) into multiple contexts, including EHR systems and research data collection. An overview of the development and instantiation processes are shown in Figure 2. Using well-defined structured data improves research processes such as variable identification, data capture, measurement methodology, and the subsequent reliability and validity of the data obtained. Well-defined data are associated with improved analytic outcomes, because the greater precision and accuracy yields greater relational specificity among identified variables in a statistical model.

Just as the taxonomy facilitates identification of data domains and data elements for medication adherence-related sciences, the process matrix facilitates selection of domains and elements for specific research questions. Selection of the optimal data element is often determined by the context in which the study is conducted and by the feasibility of obtaining a metric in a setting or patient population. For each research question, the process matrix shown in Table 1 facilitates critical thinking about options for data element access and selection. These options are based on the established study endpoints and further defined by the context of the study sample and setting. For example: study purpose and scope; study design, sample, and setting; timing of measurement; procedures; and study analysis plan all describe functional components of the research question and can be used to identify data domains and systematically select the most appropriate data elements.

**Figure 3:**
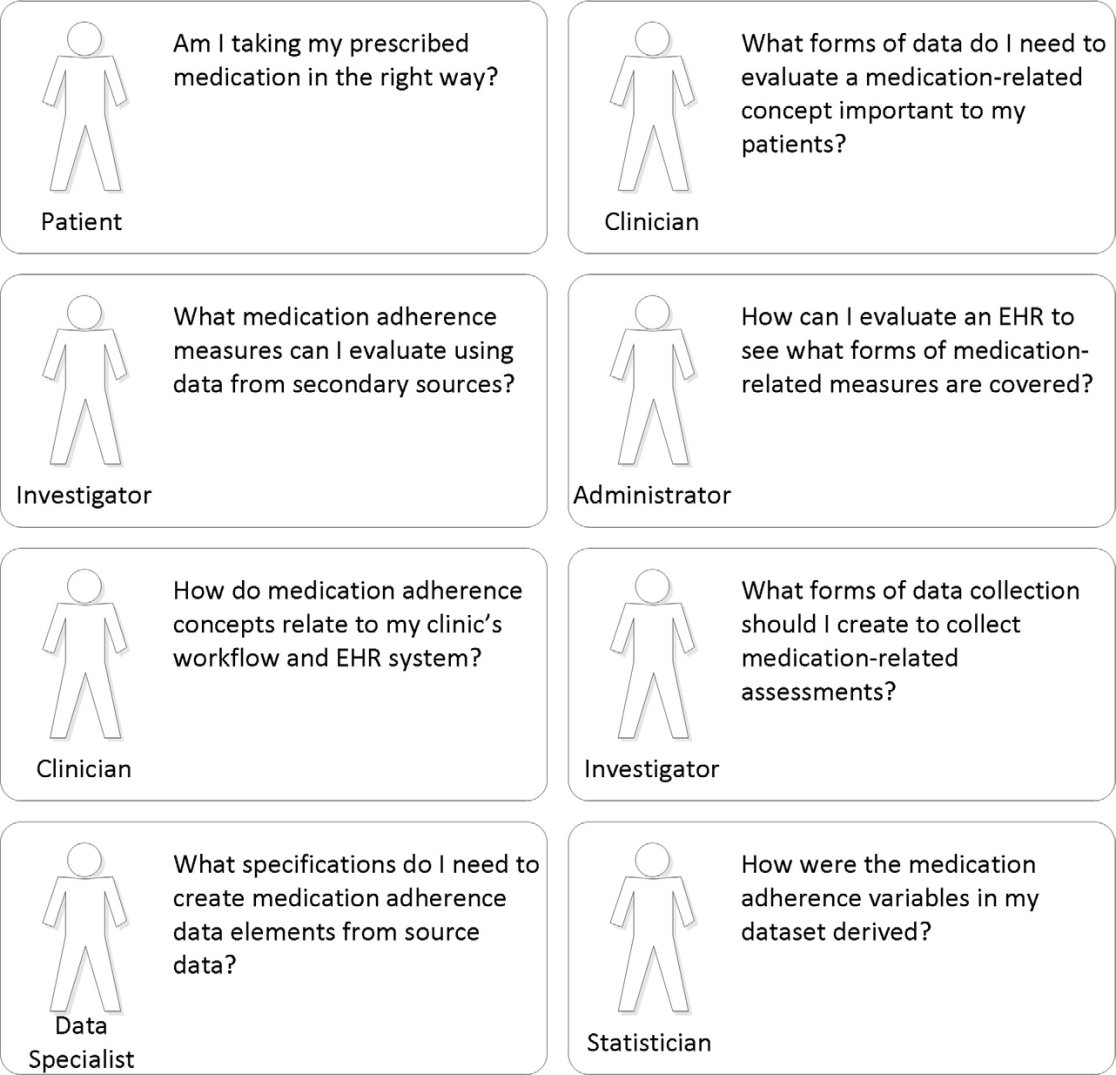
**Actor diagram**.

To demonstrate how the medication adherence data model facilitates research from the perspective of key stakeholders, patients, providers, healthcare systems, and industry, the following section describes use of the process matrix to complement the stakeholder scenarios presented in Figure 3.

### Application of the process matrix methodology for research

The first step for using the process matrix is to clarify key research goals. What is the dependent variable or primary outcome of interest? Is adherence the primary outcome (dependent variable) or a contributor (independent variable) to a more relevant primary outcome, such as serious clinical events or mortality? Choosing domains and data elements requires thoughtful evaluation of the project objectives and data accessibility. The value of adherence lies in evidence that taking the medication yields a valuable clinical outcome.

For example: taking statins decreases serious clinical events and mortality in patients with diabetes (Fallis et al., 2013). Yet the extent to which medication-taking actually occurs is < 50%, and this poor adherence contributes to the current failure to improve these important clinical outcomes. The data model therefore identifies possible standardized data options for studying medication adherence. The process matrix is designed to optimize data element selection for medication adherence as a moderator, or intermediate dependent variable, for clinical events and mortality as key clinical outcomes in the defined population (e.g., patients with type 2 diabetes). To accomplish the first step in the process matrix, identify the endpoint or dependent variable of interest and consider the role of medication-taking in relation to that primary endpoint.

For another example, consider the question: “What is the relationship between adherence to metformin (as measured by hemoglobin A1c < 6) and incidence of myocardial infarction in patients with type 2 diabetes?” The following key questions arise: How much adherence? How much of the time? To all medications or just some? Adherence by what measure? Answering these questions and using a process matrix to evaluate available data elements can yield greater specificity in the actual terms and variables used in the research question. A more measurable research question, if longer term clinical events are not available, may be: “What is the relationship between adherence to metformin or oral agents and levels of hemoglobin A1c at 6, 12, and 24 months in patients with type 2 diabetes?”

In this example, each of these questions can be answered by the data elements and definitions in the taxonomic construct *Adherence to Medications*. However, if the objective is to improve adherence, and the research question is “what factors influence/improve medication adherence?” then the second construct (*Management of Adherence*) becomes a central source for considering and selecting data elements.

The second step in using the process matrix after choosing the primary endpoint is to choose the domains and respective data elements. Data elements must be specified or selected for each domain in a research question. Therefore, as shown in Figure 3, data elements must be selected for independent variables as well as the primary dependent variable in the study. An endpoint or dependent variable (e.g., stroke) will require selection of several independent variables, one of which will be blood pressure. However, there are multiple data element options for blood pressure: systolic blood pressure, diastolic blood pressure, mean arterial blood pressure, and others. The process matrix can facilitate selection of specific, accessible data elements with standardized definitions, making study findings more generalizable and valuable to health systems, patients, providers, and researchers.

Choosing data elements and data definitions is a context-driven selection process. “Data element” refers to the most atomistic level of the data being described. Rather than “blood pressure,” data elements should be specified as described above (i.e., as systolic, diastolic, or mean arterial pressure). The information implicit in the label or name of each data element defines that element. For example, if the data element were “prescribed medication frequency,” permissible values for that data element might be: 1 ×/daily, 2×/daily, every other day, or “as needed.” Likewise, “medication name” should be specified as brand name, generic name, or simply “drug class.”

The *context* of the variable is determined by the mode of data collection. For example, medication frequency could refer to prescribed frequency or actual frequency of intake, and could be measured using self-report, MEMS cap data, or an EHR value. Selecting the data definition from among these options depends on the setting and context of the data element within the domain and the larger context of the study.

The logistics of data collection within a study design depend upon the context in which data will be collected. For example, data collection may be active (such as prospectively asking patients to bring pill bottles to a clinic visit and counting the pills) or passive (such as within the context of continuous or secondary use that utilizes previously collected data in an EHR or enterprise data warehouse). In the passive context, recognizing the domain as well as the individual data elements becomes even more important. Patient-reported medication lists and doses and dosing frequency often differ from medications prescribed and documented in computerized physician order entry (CPOE) systems in EHRs; yet each of these contexts may have similar data elements.

Research on medication adherence in the context of population-based interventions designed to improve quality, outcomes, and cost requires careful specification of data elements across all domains. The process matrix can be used to consider variable access and availability in passive EHR data systems. It can also be helpful in prioritizing active data collection efforts to obtain the most important incomplete data elements for key research questions.

## Discussion

In the course of our initial attempts to devise a framework for capturing medication adherence data in the setting of a population-based intervention, we realized existing EHR data sources in the four participating counties were insufficient for research aimed at supporting the aims of improved quality, clinical outcomes, and costs in patients with diabetes. To identify key indicators of medication adherence and management of adherence, we applied taxonomic constructs (and their accompanying data domains, elements, and definitions) and a process matrix methodology to ensure data accessibility. Using this structured approach to identify pre-specified variables amplified the wide gaps in EHR data capture for the data elements reflecting both *Medication Adherence* and provider and health system support for *Management of Adherence*.

Lack of data connectivity and reciprocity across settings of care delivery has been identified as a key factor in poor data capture (Kush et al., 2007; Abernethy et al., 2010). Behavioral strategies to support medication-taking in this population were broadly implemented through clinics, community venues, and patient home visits, yet EHRs in each of the four county sites lacked designated fields for documenting participation in these interventions. This oversight rendered impossible the task of measuring and collecting feedback regarding the impact of these care processes, and key stakeholders lacked data to support effective communication regarding participation in adherence interventions. For example, the Stanford Chronic Disease Self-Management Program (CDSMP) (Lorig et al., 2001), which was offered in short sessions once a week for 6 weeks in community settings such as churches, senior living facilities, and community education centers, was not represented in data capture. Patient participation in these programs was difficult to ascertain from EHRs.

Because data capture was absent or, at best, available only in the isolated setting in which the program was offered, progress toward targeted adherence goals was difficult to measure. Actual patient data could not be included in communications between patients and providers to support program participation or the need for redirection. Thus, the quality of care improvement initiatives failed to provide key metrics that could affect clinical endpoints, and existing sources of data such as EHRs fail to capture important measures of research quality data for adherence-related variables.

A second major barrier to data capture was the inability to effectively use the national drug code (NDC) information (U.S. Food and Drug Administration, 2013) from the various EHR and claims datasets across each county system for adherence purposes. Though NDC codification provides a unique 10-digit drug identifier for each FDA-approved medication, the 3-segment numeric indicator of the vendor, product specification (strength, dose, and formulation of the drug), and trade package are not standardized. Only the first segment is a fixed identifier from the FDA, while the second and third segments vary by company and product. As a result, although NDC codification is intended to greatly improve data quality through coded, standardized identifiers, the stage at which the codes are implemented in the EHR system is critical. For example, all health systems in the four-county project use medication data collection that starts with patient-reported, free-text medication names. Codification and drug mapping occur later, after the data are collected. Although this is logical for “usual care,” it is less precise and requires more processing. When NDC codes are captured at point of dispensation or provider ordering in the EHR, the data quality gap is closed and drug adherence tracking to the community pharmacy level is possible. This level of sophistication in use of NDC codes is not yet widely available, and this lack contributes to the gap in data quality and accessibility.

### The opportunity to advance the science of adherence using the taxonomy

The application of the adherence taxonomy for research provides a rich opportunity for identifying and selecting standardized measures and metrics for a wide variety of research questions and study designs. The value lies in specifying and standardizing definitions, measures and metrics for each of the overarching constructs of the taxonomy. This step lays the foundation for subsequent work to integrate these data element definitions and standards across electronic data platforms that include pharmacies, EHRs, and insurance claims databases (Raebel et al., 2013) as well as electronic data platforms of associated care delivery devices such as telephone and home technology devices. Other settings where patients receive support for management of adherence, such as health departments, dialysis clinics, retail stores, and community-based healthcare education settings must also be able to share standardized data definitions and measures if we are to advance the science of management of adherence as a data-driven construct.

In our four-county samples, the variation in data reflecting medication management was staggering. For example, in education plans, care plans, and methods of evaluation chosen for each of these patient-provider consultations, what was measured and documented about medication-taking varied substantially. Likewise, the specification of role-responsibilities for supporting the management of adherence across participant groups including patients, caregivers, providers, health systems, and community-based resource groups, was highly variable and not accessible for analysis in existing electronic platforms. Our findings suggest opportunities to specify standard measures for management of adherence to reframe medication-taking as a behavior, one in which all actors—patients, caregivers, providers, health systems, and communities—play a measurable role. Doing so changes the culture of management of adherence by recognizing needed shifts in responsibilities for the work of adherence, and by reflecting those shifts in the data that are captured for evaluation. Applying the taxonomy through use of electronic data capture systems has the added potential to create consistent, standardized measures of self-management and support strategies across population of patients, providing the opportunity for useful comparative effectiveness studies on management of adherence in the future.

### Benefits to patients, providers, healthcare systems, and industry

The need exists to conceptually map data domains and elements, as well as data sources embedded in care delivery processes that contribute to medication adherence on a larger scale. Such a map would lend structure and definition to the data capture required for innovative study designs and would incorporate the complex relationships between individuals, providers, health system and environmental factors that contribute to medication adherence. In addition, the map would allow for more systematic approaches to study broad-scale adherence interventions and to evaluate the relationship of improved adherence to clinical events and mortality across patient populations.

A conceptual model has rarely held tangible value for patients, clinicians or the financially driven business of healthcare systems or the pharmaceutical industry. Yet in this case the model provides a schematic diagram to guide movement in both practice and research to identify measureable indicators of management of adherence. By measuring key contributing factors from each of the players in the adherence game, data-driven solutions for improvement may be more readily identified. The data model allows measurement of life choices, behaviors, resources and support structures to become more clearly visible. Integrating process models that reflect management of medications across everyday life, healthcare delivery and access with data models that reflect the data sources and data elements at each process step enables the conceptual data model to effectively map opportunities for improving through visibility of meaningful, real-time data (Payne et al., 2009).

Such a data model would benefit patients and providers by informing care delivery in meaningful way; it accounts not only for the patient and the pills, but also for the complexity of the broader landscape of social determinants of health, including family, community, access to information technology, and health system support. Such a data model may provide a roadmap to guide the transition of adherence evaluation from the controlled world of clinical trials to the messiness of population-based settings that capture “big data” by using EHRs, claims data, or community-level studies that evaluate thousands of people in real time, as in the case of the SEDI project. This concept was aptly illustrated by kent (1978), who observed that in the real world,”… highways are not painted red, rivers don't have county lines running down the middle, and you can't see contour lines on a mountain. The task of the data modeler is to create order out of chaos without excessively distorting the truth.” As we have observed in designing data collection for the SEDI project, current approaches to population-based evaluation of adherence fall short of the truth by missing critical opportunities for data capture. As a result, the opportunity is lost to effectively harness data reflecting key principles of the behavioral economics of medication-taking.

## Conclusions

We propose that the approach developed for operationalizing the taxonomic domains for *Management of Adherence* in the SEDI project will allow more effective analysis and will more accurately depict the messiness of the real world by mapping the relationships among disparate contributors to medication adherence and describing their relative contributions to the goals of achieving improved quality, clinical outcomes, and costs. This formative work suggests that opportunities exist to further develop and test a broad data model for adherence—one that depicts a detailed roadmap of avenues for variable selection related to adherence and management of adherence.

## Author contributions

Dr. Granger created the initial draft of the manuscript. All authors revised subsequent drafts of the manuscript and contributed to analysis and interpretation of the data. All authors read and approved the final version of the manuscript for submission.

### Conflict of interest statement

Drs Califf and Spratt have the following disclosures. A complete and continuously updated list of disclosure information for Dr. Califf is available at https://dcri.org/about-us/conflict-of-interest. Conflict of Interest: Dr. Granger, Ms. Rusincovitch, Ms. Avery, Dr. Dunham, Dr. Batch, Dr. Feinglos, Ms. Kelly, and Ms. Pierre-Louis have no conflicts of interest to report. Dr. Spratt reports relationships with Frederick O'Connor Medical Consultants and The Exeter Group. Dr. Califf reports receiving research grants that partially support his salary from Amylin, Johnson & Johnson, Scios, Merck/Schering-Plough, Schering-Plough Research Institute, Novartis Pharma, Bristol-Myers Squibb Foundation, Aterovax, Bayer, Roche, Lilly, and Schering-Plough; all grants are paid to Duke University. Dr. Califf also consults for TheHeart.org, Johnson & Johnson, Scios, Kowa Research Institute, Nile, Parkview, Orexigen Therapeutics, Pozen, WebMD, Bristol-Myers Squibb Foundation, AstraZeneca, Bayer/Ortho-McNeil, Bristol-Myers Squibb, Boehringer Ingelheim, Daiichi Sankyo, GlaxoSmithKline, Li Ka Shing Knowledge Institute, Medtronic, Merck, Novartis, Sanofi-Aventis, XOMA, University of Florida, Pfizer, Roche, Servier International, DSI-Lilly, Janssen R&D, CV Sight, Regeneron and Gambro; all income from these consultancies is donated to non-profit organizations, with most going to the clinical research fellowship fund of the Duke Clinical Research Institute. Dr. Califf holds equity in Nitrox LLC, N30 Pharma, and Portola. Full listings of Dr. Califf's relationships with industry are available at http://www.dukehealth.org/physicians/robert_m_califf and at https://dcri.org/about-us/conflict-of-interest.
